# Construction of a new automatic grading system for jaw bone mineral density level based on deep learning using cone beam computed tomography

**DOI:** 10.1038/s41598-022-16074-w

**Published:** 2022-07-27

**Authors:** Yanjun Xiao, Qihui Liang, Lin Zhou, Xuezhi He, Lingfeng Lv, Jiang Chen, Su Endian, Guo Jianbin, Dong Wu, Lin Lin

**Affiliations:** 1 Fujian Key Laboratory of Oral Diseases and Fujian Provincial Engineering Research Center of Oral Biomaterial and Stomatological Key Lab of Fujian College and University, School and Hospital of Stomatology, Fujian Medical University, Fuzhou, Fujian China; 2 Newland Digital Technology Co., Ltd., Fuzhou, Fujian China; 3 Fujian Provincial Engineering Research Center of Oral Biomaterial, School and Hospital of Stomatology, Fujian Medical University, Fuzhou, Fujian China; 4 Institute of Stomatology and Research Center of Dental and Craniofacial Implant, School and Hospital of Stomatology, Fujian Medical University, Fuzhou, Fujian China; 5 Stomatological Key Laboratory of Fujian College and University, School and Hospital of Stomatology, Fujian Medical University, Fuzhou, Fujian China; 6 Fujian Key Laboratory of Oral Diseases, Fujian Medical University, School and Hospital of Stomatology, Fujian Medical University, Fuzhou, Fujian China; 7 Research Center of Dental and Craniofacial Implants, School and Hospital of Stomatology, Fujian Medical University, Fuzhou, Fujian China

**Keywords:** Computer science, Medical research

## Abstract

To develop and verify an automatic classification method using artificial intelligence deep learning to determine the bone mineral density level of the implant site in oral implant surgery from radiographic data obtained from cone beam computed tomography (CBCT) images. Seventy patients with mandibular dentition defects were scanned using CBCT. These Digital Imaging and Communications in Medicine data were cut into 605 training sets, and then the data were processed with data standardization, and the Hounsfiled Unit (HU) value level was determined as follows: Type 1, 1000–2000; type 2, 700–1000; type 3, 400–700; type 4, 100–400; and type 5, − 200–100. Four trained dental implant physicians manually identified and classified the area of the jaw bone density level in the image using the software LabelMe. Then, with the assistance of the HU value generated by LabelMe, a physician with 20 years of clinical experience confirmed the labeling level. Finally, the HU mean values of various categories marked by dental implant physicians were compared to the mean values detected by the artificial intelligence model to assess the accuracy of artificial intelligence classification. After the model was trained on 605 training sets, the statistical results of the HU mean values of various categories in the dataset detected by the model were almost the same as the HU grading interval on the data annotation. This new classification provides a more detailed solution to guide surgeons to adjust the drilling rate and tool selection during preoperative decision-making and intraoperative hole preparation for oral implant surgery.

## Introduction

Clinically, there is no clear consensus on the definition of bone quality, but in general it covers many aspects such as the degree of bone mineralization and the shape and type of the bone trabecula. At present, the most widely used jaw bone quality classification is the four types of jaw classification proposed by Lekholm and Zarb in 1985^1^, in which the jaw bones are divided into one of four types according to the amount of cortical bone and cancellous bone in the radiological images of the jaw^2^: Type I bone is considered the least vascular and most homogenous, type II is a combination of cortical bone with a marrow cavity, type III is predominantly composed of trabecular bone, and type IV is described as having a very thin cortex and low-density trabeculae. However, the existing grading method is limited to the grading of bone quality. This is a disadvantage in the context of implant surgery, where it is common to observe bones of different densities in one area. Thus, using this classification system alone may cause offset of the implant site or the loss of torsion during implant placement. We believe that the ultimate goal of bone classification in oral implant surgery is to guide a more comprehensive understanding of the implant site area, so as to help the surgeon decide the size of the cavity preparation and the choice of implant diameter during the implant surgery. In addition, an improved bone classification system will help to improve the initial stability of the implant and the resistance to drilling during the operation.

In this study, a deep learning artificial intelligence method was adopted to determine the bone density types at different locations of the implant sites from the radiological data obtained from cone beam computed tomography (CBCT) images, so as to provide a more detailed bone density classification range. This method can also improve the accuracy of bone mineral density grade judgment and reduce the possibility of errors in individual subjective judgment. Based on this method, an automatic classification method was developed and verified, and a new classification standard of jaw bone was proposed. The jaw bone is divided into five types, type 1 being the densest, and type 5 being the loosest. The range of bone types is automatically calibrated through the use of a big data artificial intelligence system to better guide clinicians in implant surgery operations.

## Material and methods

**Figure 1:**
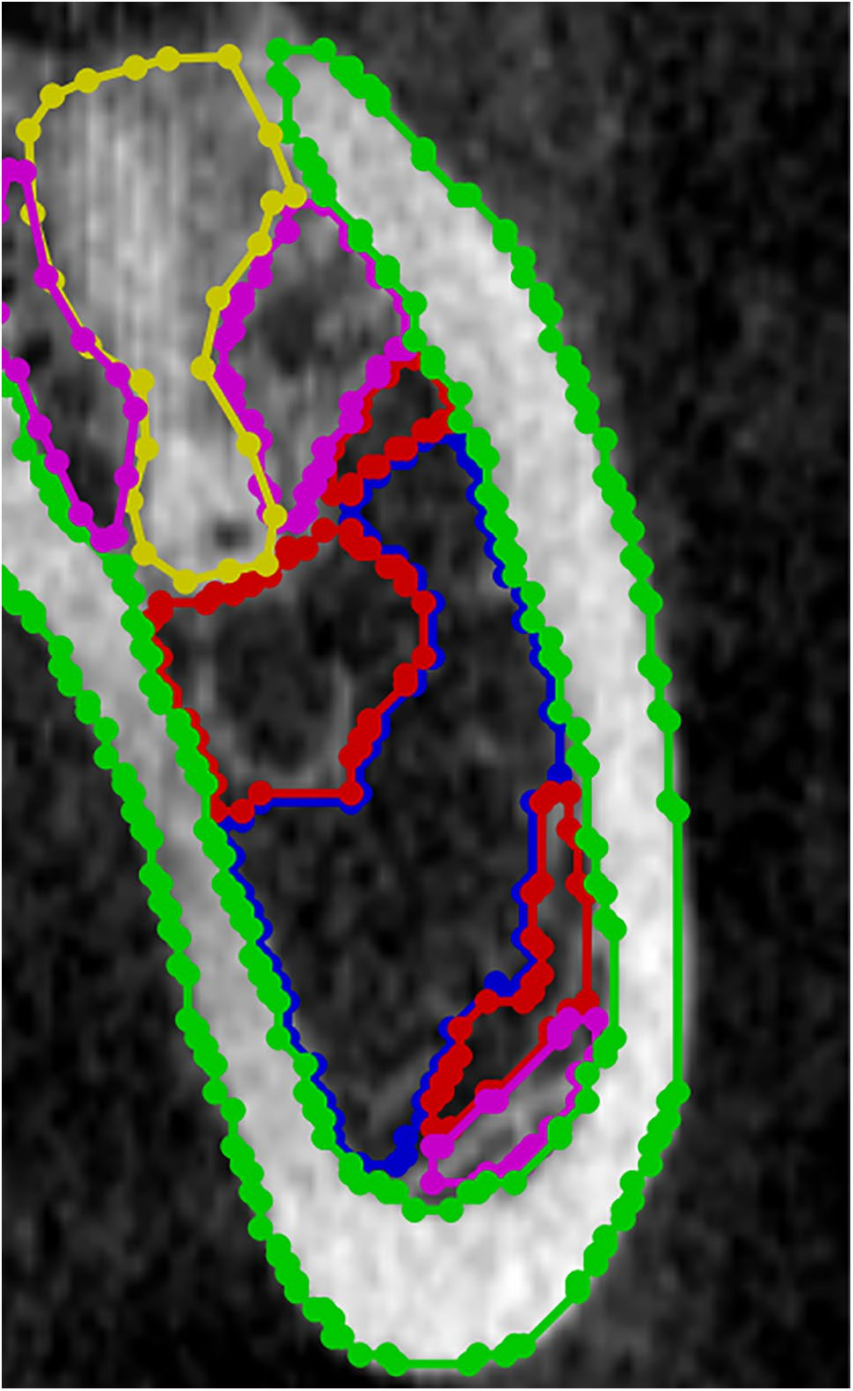
Bone density annotation picture.

**Figure 2:**
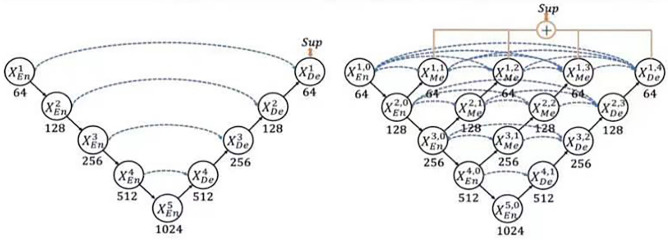
Model structure of UNet and Nested-UNet.

1. Data labeling and processing: Seventy patients with mandibular dentition defects who were treated at the Affiliated Dental Hospital of Fujian Medical University from March 2020 to September 2020 were selected and scanned with CBCT (KAVO i-CAT). The 70 DICOM data were extracted and imported into the preprocessing program for vertical sagittal arch cutting, with about 10 pieces cut per dataset, and finally got 605 training sets. The recognized types of jaw bone, according to the HU values, were as follows: Type 1, 1000–2000; type 2, 700–1000; type 3, 400–700; type 4, 100–400; and type 5, − 200–100. Data standardization was performed first. From experience, HU values that fell within the range of − 200–2000 were extracted. Those higher than the maximum value were set as the maximum value, and those lower than the minimum value were set as the minimum value. For the convenience of labeling, DICOM data mapping was converted into PNG format and imported into the graphical image annotation tool LabelMe 4.5.6. Four trained dental implant physicians combined the HU values of the slices to manually identify and label the area of the jaw bone density level in the image (Fig. 1). Then, a physician with 20 years of clinical experience confirmed the labeling level using the HU value of the LabelMe software as a guide. The random selection of images was repeated five times to confirm that the classifications were correct. Moreover, each evaluator was compared to the reference standard classification to evaluate the effectiveness of subjective classification. The datasets were divided into training sets and test sets at a ratio of 9:1.
2. Model construction: In the current study, we chose Nested-UNet^3^ as the backbone to extract and fuse multiscale information to determine the corresponding semantic segmentation results. Nested UNet involves stacking different levels of UNet^4^, in which the addition of dense short joins (upper and lower sampling) serves to achieve a better combination of depth and shallow features and better feature extraction performance than UNet (Fig. 2).

Based on Nested U-net, we optimized the corresponding loss using the “Pixel-Level” of “FocalLoss”^5^ (1) and the Class-Level of loss Diceloss (2), and then two loss according to certain weight (3) the total loss resulting from the weighted function. Using Diceloss alone will reduce the training stability. The addition of FocalLoss can resolve the imbalance of positive and negative samples and accelerate convergence. Optimization through two different dimensions of loss can also help the model better understand the task.

1 $${\text{Focal}}\;loss = \left\{ {\begin{array}{*{20}c} { - \alpha {(1} - {\text{y}}^{\prime } {)}^{\gamma } \log y^{\prime } ,y = 1} \\ { - (1 - \alpha )y^{\prime \gamma } \log (1 - y^{\prime } ),y = 0} \\ \end{array} } \right\}$$

1. In Eq. 1, y represents the label and y’ represents the prediction results. The balance factor $\alpha$ is used to balance the uneven ratio of positive and negative samples. The coefficient $\gamma$ can reduce the loss of easy-to-divide samples. Focus on difficult samples.
  2 $$D{\text{ice}}\;loss = {1} - \frac{2|A \cap B|}{{|A| + |B|}}$$
2. A and B represent the predicted result and Ground Truth (GT), respectively. $\left| {A \cap B} \right|$ is the intersection of AB, and $\left| A \right|\left| B \right|$ represent the number of elements of A and B, respectively.
  3 $$L = \alpha L{\text{focal}}\;{\text{l}}oss + \beta Ldice\;loss$$

In addition, we optimized the data enhancement module, and performed operations such as random rotation and scaling of the data to improve the robustness of the model. Finally, the segmentation results were processed by the corresponding connected domains to obtain the final segmentation results.

### Ethical approval

This study was performed in line with the principles of the Declaration of Helsinki. Approval was granted by the Ethics Committee of Biomedical Research of the Affiliated Stomatological Hospital of Fujian Medical University (Date 8.9.2021/No.60).

### Informed consent

Informed consent was obtained from all individual participants included in the study.

## Result

After the model was trained on 605 training sets, the average dice of each category on 68 test sets could reach a maximum of 0.75.

The 68 test sets were counted, and the statistical results of the HU mean labeled by physicians, and the HU mean detected by the model are shown in Table 1. It can be seen that the mean HU of all categories in the neural network test results is almost the same as the HU grading interval labeled by physicians.

**Table 1:** The mean HU of all categories of physicians labeled and model detected.

Type	1	2	3	4	5
Standard HU value interval	1000–2000	700–1000	400–700	100–400	− 200–100
HU mean labeled by physicians	1519	920	693	351	195
HU mean labeled by model prediction	1520	964	648	352	136

The results of analysis and comparison between the standard deviation of the HU value labeled by physicians and the standard deviation predicted by the model are shown in Table 2. It can be seen that when the model predicted each category, the standard deviation of the HU value in this category was consistent with the standard deviation of the category labeled by physicians. This indicates that the model has learned that the HU value of each bone mineral density level should fluctuate within a certain range without excessive deviation.

**Table 2:** The standard deviation of HU values of all categories marked by physicians and the model prediction results.

Type	1	2	3	4	5
HU standard deviation labeled by physicians	350	309	359	307	303
HU standard deviation predicted by the model	369	327	371	315	286

**Figure 3:**
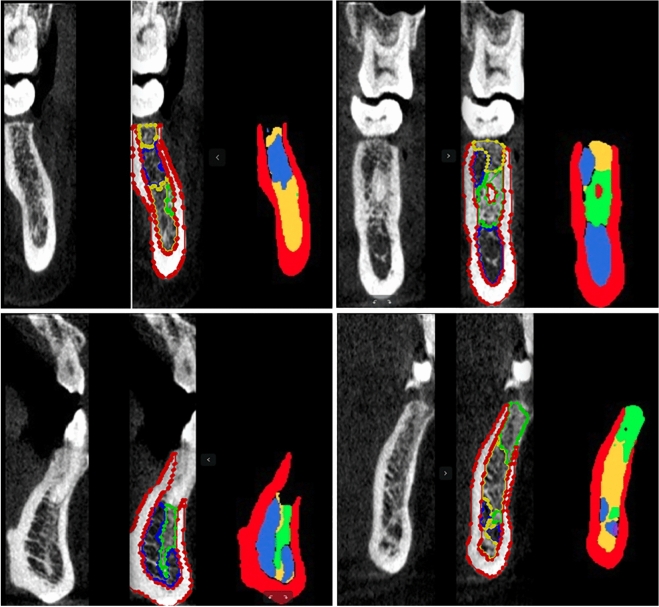
Bone mineral density section and its identification effect map. From left to right are the original image, doctor’s label and model identification effect map.

The recognition effect is shown in Fig. 3 (types 1–5 are represented by red, yellow, green, blue, and purple, respectively).

## Discussion

### Advantages of artificial intelligence and its application in stomatology

Within artificial intelligence (AI), machine learning has emerged as the method of choice for developing practical software for computer vision, speech recognition, natural language processing, robot control, and other applications^6^. As decisions are made based on the combination of computer processing of data and algorithms, AI can improve accuracy and reduce the chance of errors compared to humans in the same situation. In addition, unlike human beings, machines are not affected by subjective factors such as emotional factors, mental state, and personal experience, so the efficiency of machines when dealing with problems is greatly improved, which enables correct decisions to be made quickly. The combination of AI such as in the context of diagnosis and treatment procedures, can greatly reduce the risk of misdiagnosis.

Artificial intelligence has been extensively studied in the field of dentistry. Indeed, Lu et al.^7^ analyzed samples from 36 patients with head and neck tumors using artificial intelligence deep learning, and constructed an artificial intelligence model using hyperspectral imaging technology. This technology can predict the boundary of head and neck tumors with an accuracy of up to 91%, which is significantly better than traditional fluorescence imaging technology. In another artificial intelligence system focused on early detection of tumors, Uthoff et al.^8^ combined fluorescence imaging technology with artificial intelligence to develop an early prediction device for oral tumors. By collecting natural images and fluorescence imaging images of intraoral tissue, combined with AI, patients with early cancer can be identified quicker and easier, with a prediction accuracy of up to 80%. In the field of stomatology, the Japanese scholar Hiraiwa et al.^9^ used the imaging data of 760 mandibular first molars in an artificial intelligence model to predict the presence of double distal roots with an accuracy of 86.9%. In addition, there are also extensive studies in the application of artificial intelligence in the prediction of periodontal lesion state, tumor lymph node metastasis prediction, and auxiliary colorimetry in aesthetic repair.

The use of artificial intelligence combined with big data allows researchers to provide a snapshot of the real world from population-level clinical data. In addition, as a powerful data network connection, previously unrelated isolated datasets between different fields are integrated with big data to provide new possibilities for the discovery of biological manifestations, research progress, and clinical associations of diseases^10^.

### Issues with the existing bone classification

At present, the most widely used jaw grading was proposed by Lekholm and Zarb in 1985^1^, in which the grading is divided into four categories according to the proportion of compact bone and spongy bone. However, this classification method is not sufficiently accurate, which is reflected by the difficulty in distinguishing between type II bone and type III bone^11^. In addition, this classification method is limited to the classification of bone quality, which is based on the whole classification of the jaw block, and does not reflect the bone condition of the local part of the jaw or other specific sites.

In 2018, Asama et al.^12^ proposed a revised L&Z (Lekholm and Zarb) classification, which considered all possible combinations of compact bone and spongy bone. Although compact bone and spongy bone can be clearly distinguished with high repeatability, it is not sufficient to directly guide the implantation procedure.

In 1994, Klemetti et al.^13^ divided the mandible into three categories based on the X-ray morphology of the lower margin of the mandible on the oral surface slice: Cl, the endosteal margin of the cortex was even and sharp on both sides; C2, the endosteal margin showed semilunar defects (lacunar resorption) or seemed to form endosteal cortical residues (one to three layers) on one or both sides; and C3, the cortical layer formed heavy endosteal cortical residues and was clearly porous. Statistical studies of large datasets demonstrated that there is a positive correlation between the mineral density of bones and the changes in the mandibular cortex. Yet, panoramic images provide too little information to definitively diagnose the risk of osteoporosis.

Later, Nicolielo et al.^14^ developed a computer-based automatic bone classification method. According to the trabecular bone parameters obtained by CBCT, all bone regions were classified into three trabecular pattern classes (sparse, intermediate, and dense), and morphometric parameters were used to automatically classify the trabecular patterns. This method has higher retest consistency and reliability. However, the proposed classification is relatively general and requires artificial follow-up analysis before implantation.

Some scholars classify the jaw according to the hand feel during the drilling process. Greenstein et al.^15^ divided the jaws into four types based on the tactile feedback from the 2 mm twist drill: D1 feels like drilling into oak or maple, D2 feels like drilling into pine or spruce, D3 feels like drilling into balsa wood, and D4 feels like drilling into Styrofoam. This method can guide the subsequent implantation operation according to the feeling during drilling. However, since most clinicians lack the experience of drilling wood with different textures, and considerable surgical experience is gained by relying on the feel, the hand feel classification is not widely accepted.

### Characteristics of the new classification and its significance in clinical application

The traditional jaw classification focuses on different bone types in different regions of the jaw, and there remains a lack of analysis of different positions in the same region. The new jaw bone classification can fill this gap to a certain extent by covering preoperative diagnostic evaluation and intraoperative decision-making to reducing the difficulty of decision-making in implantation.

The new classification divides the jaw bone density from high to low (type 1–5) according to the HU value of CBCT. Type 1 bones are the densest, suggesting that in these cases, attention should be paid to the blood supply at the implant site and the cooling during the implant preparation. Type 5 bones are the most loose, indicating that attention should be paid to the initial stability of the implant and the possibility of implant osseointegration failure in this type of bone.

The new classification system outlined here is an artificial intelligence classification system that has been designed to guide the clinical implantation decision. Artificial intelligence is used for deep learning of the model to improve the accuracy of classification. This technique has high potential to showcase the application of precision medicine in the field of oral implantology. The cornerstone of precision medicine is naturally the ability to make precise diagnoses based on a mechanistically informed taxonomy, and the consistency of results can be guaranteed using machine classification^16^. After artificial intelligence analysis determines the quality classification of the jaw bone, it can directly propose a reasonable implant process plan, which improves the accuracy of the clinical operation.

This new classification provides a more refined solution for implant surgery. In clinical practice, differences in jaw bone density are often encountered at implant sites in the jaw-gingival, mesiodistal, and buccal-lingual directions. Thus, the drill needle can easily deviate from the preoperative design position in the horizontal direction toward the less osteoporotic part and the vertical upward part. This is often due to accidental perforation caused by a sudden decrease in bone mass or a sharp increase in the temperature of the drill head caused by the increase in bone density, which consequently affects osseointegration. The traditional classification model cannot provide the surgeon with the specific distribution position of the different densities of the jawbones, which may cause the surgeon to misjudge during the implantation process. However, the new jaw classification can clearly identify the loose or dense sites in the jaw. Combined with the conventional imaging data measurement and analysis, it can guide physicians to adjust the drilling speed and the selection of drilling tools in the process of preoperative decision-making and intraoperative hole preparation. For example, in the application of computer-guided implant surgery (implant navigation surgery/template guided implant surgery), the technology can be used to indicate the density of different areas of the jaw bone, which can be used to guide the optimal three-dimensional location of the implant.

With regard to the limitations of this method, the new classification method lacks clinical prospective studies to confirm its practical feasibility. Thus, it is necessary to use a new type of jaw bone classification to assess the correlation between the initial stability of the implant and the resistance to the cavity. Therefore, further research is needed, including the use of artificial intelligence big data to investigate the epidemiological characteristics of different jaw bone types and jaw bone types in different regions in the population.

## Data Availability

The datasets analysed during the current study are available from the corresponding author on reasonable request.
